# P01.17. Development of a biocrystallisation method for examining effects of homeopathic preparations on germinating cress seeds

**DOI:** 10.1186/1472-6882-12-S1-P17

**Published:** 2012-06-12

**Authors:** S Baumgartner, P Doesburg, C Scherr, J Andersen

**Affiliations:** 1 University of Bern, Institute of Complementary Medicine KIKOM, Bern, Switzerland; 2 Crystal Lab, Ottersum, Netherlands; 3 Biodynamic Research Association Denmark, Galten, Denmark

## Purpose

A major challenge of homeopathic basic research is to develop test systems that yield consistent results. Outcome of plant bioassays is usually based on growth parameters (e.g. germination rate, seedling length, leaf area). We aimed to evaluate the potential of a crystallisation method with additives ("biocrystallisation") as a complementary outcome measure. The method used is based on the crystallographic phenomenon that when crystallising watery solutions of dihydrate CuCl_2_ in the presence of organic additions (juices/extracts), reproducible dendritic crystal structures are observed. The resulting biocrystallograms can be evaluated visually and/or by computerized image analysis.

## Methods

Cress seeds (Lepidium sativum L.) germinated and grew *in vitro* in either Stannum met. 30x or water 30x. Per experiment, six coded (blinded) 30x preparations were applied in randomized order, representing three independent replicates of the two treatments. Seedlings grew for 96 hours in darkness and were subsequently processed into a watery extract. Biocrystallisation was performed on circular glass plates in 6-fold replication per treatment group, yielding 36 biocrystallograms per experiment. A total of 15 independent experiments were performed at two independent laboratories. Biocrystallograms were scanned and analysed by computerized texture image analysis, using 15 second-order parameters as outcome measure. 3-way-ANOVA with the independent parameters treatment (n=2), internal replicate (n=3), and number of experiment (n=15) was used to analyze the data.

## Results

All 15 texture analysis variables yielded significant or highly significant results for the homeopathic treatment. Two variables yielded differences between the internal replicates, most probably due to a processing order effect. There were only minor differences between the results of the two laboratories.

## Conclusion

The texture of biocrystallograms of homeopathically treated cress exhibited specific characteristics, differentiating water 30x and Stannum met. 30x. Thus, the biocrystallisation method seems to be a promising complementary outcome measure for plant bioassays investigating effects of homeopathic preparations.

